# Perception of disease, dyadic coping, and the quality of life of oncology patients in the active treatment phase and their life partners: an approach based on the actor-partner interdependence model

**DOI:** 10.3389/fpsyg.2023.1069767

**Published:** 2023-04-27

**Authors:** Adelina Mihaela Ştefănuţ, Mona Vintilă, Larisa Maria Bădău, Daciana Grujic, Cristina Marinela Oprean, Cosmin Goian, Paul Sârbescu

**Affiliations:** ^1^Department of Psychology, Faculty of Sociology and Psychology, West University of Timişoara, Timişoara, Romania; ^2^Hygiene Department, Victor Babeș University of Medicine and Pharmacy, Timișoara, Romania; ^3^Department of Plastic and Reconstructive Surgery, Victor Babeș University of Medicine and Pharmacy, Timișoara, Romania; ^4^Morpho-pathology Department, Victor Babeș University of Medicine and Pharmacy, Timișoara, Romania; ^5^Department of Social Assistance, Faculty of Sociology and Psychology, West University of Timişoara, Timişoara, Romania

**Keywords:** cancer, illness appraisal, dyadic coping, quality of life, QoL, APIM

## Abstract

**Objective:**

The aim of this study based on the Systemic Transactional Model was to examine the relationship between dyadic coping and (1) disease perception and (2) quality of life of a sample of cancer patients and their life partners.

**Method:**

This cross-sectional study included 138 oncological dyads. The following questionnaires were used: Stress Appraisal Measure, Dyadic Coping Inventory, and European Organisation for Research and Treatment of Cancer QLQ-C30. Data collected was analysed by applying the actor-partner interdependence model.

**Results:**

The perception of the disease as a threat as well as its centrality significantly negatively influences the positive forms of dyadic coping whilst the perception of the disease as a challenge has a significant positive influence on them. Dyadic coping does not influence symptoms but has significant influences on global health/quality of life.

**Conclusion:**

This study has highlighted new information regarding how couples cope with cancer. The results encourage the inclusion of the perception of the disease and dyadic coping in interventions that aim to improve the quality of life of cancer patients and their life partners.

## Introduction

A cancer diagnosis is a major stress factor for both patients and their families. Patients often face depression, anxiety, and fear concerning disease progression and death (Syrjala et al., 2004). About one-third of patients report clinical levels of distress, adaptive disorders, post-traumatic reactions and fear of disease recurrence (Zabora et al., 2001; Stein et al., 2008). As in the case of other diseases, cancer patients often report sleep disorders (Davidson et al., 2002; Leuci et al., 2022), 25%–59% of them facing sleep disturbances (Howell et al., 2014) which in turn are associated with mental health problems such as depression (Koopman et al., 2002) and anxiety (Mystakidou et al., 2005). All of this is reflected in the low quality of life of oncology patients, such as those diagnosed with breast cancer (Perry et al., 2007; Montazeri et al., 2008), prostate cancer (Davis et al., 2001), ovarian cancer (Bodurka-Bevers et al., 2000; Tuncay, 2014), lung cancer (Henoch et al., 2007), and cancers of the head and neck (Van den Berg et al., 2008).

Although the whole family is disrupted when one member is diagnosed with the disease, the ones most affected are those who take the leading role in support and care, and these are most often the life partners/spouses of the patients (Hagedoorn et al., 2008; Kim and Spillers, 2010). Involved in complex care tasks for which they have little training or support (Given et al., 2001), faced with the possibility of losing a loved one, life partners of oncological patients report negative consequences for physical and mental health (Vintilă et al., 2019), a high level of distress (Pinquart and Duberstein, 2005; Moreira and Canavarro, 2013), sometimes even higher than that of patients (Couper et al., 2006). Studies have shown not only that the partners of oncology patients have a poor quality of life (QoL; Nijboer et al., 1998; Kershaw et al., 2004; Tuinman et al., 2004; Kitrungrote and Cohen, 2006; Wagner et al., 2006), but also that there is a link between patient QoL and their partners’ QoL (Patterson et al., 2013; Duggleby et al., 2015). In addition, an interdependence has been found between patients’ and partners’ well-being: Chen et al. (2004) state that the social and functional aspects of patients’ QoL have a significant influence on their partners’ QoL, and Kornblith et al. (2001) state that an increase in the symptomatology of prostate cancer patients is followed by a decrease in the QoL of their partners.

Such results have led to cancer to be considered a “we-disease” (Kayser et al., 2007) which had as a consequence the development of psychological interventions dedicated to couples and not only to their members, patients or partners. Such interventions have focused, for example, on restoring equity in the couple and encouraging the acceptance of social support (Kuijer et al., 2004), developing communication skills and changing stressful communication patterns (Kalaitzi et al., 2007; McLean et al., 2013), and included elements of existential and cognitive behavioural psychotherapy (Couper et al., 2015). Following these interventions, significant results were obtained in improving the quality of the relationship (Kuijer et al., 2004; McLean et al., 2013), the quality of life (Couper et al., 2015) as well as in reducing depression (Kuijer et al., 2004; Kalaitzi et al., 2007; Hsiao et al., 2016) and anxiety (Kalaitzi et al., 2007).

In this context, dyadic coping refers to how patients and their life partners interact to cope with the disease (Berg and Upchurch, 2007). This study, based on the Systemic Transactional Model (Bodenmann, 2005), aims to increase understanding of how dyadic coping is associated with the perception of disease and quality of life in patients undergoing active treatment and their life partners.

The Systemic Transactional Model starts from the Transactional Stress Theory of Lazarus and Folkman (1984) and extends it systemically. Thus, Bodenmann (2005) states that the stress perceived by one partner is communicated verbally or non-verbally to the other partner, and the latter interprets it and engages in a form of dyadic coping that can be positive or negative. *Positive dyadic coping* comprises three types of strategies: (1) *supportive dyadic coping*, which refers to assistance provided to the partner in his or her coping efforts (empathic understanding); (2) *delegated coping*, whereby one of the partners takes over the responsibilities of the other to help him or her; (3) *common coping*, by which the two act together to deal with the situation. *Negative coping* includes ambivalent, hostile, and superficial behaviour. The purpose of dyadic coping is to restore homeostasis both individually and at the couple level. Although dyadic coping was initially defined to take account of minor stressors in daily life, it was subsequently extended to critical life events. A cancer diagnosis can be regarded as a critical event of this kind.

In recent years, the Systemic Transactional Model has been the theoretical basis for several studies aimed at understanding how cancer patients and their life partners deal with the disease. This research has looked both at the factors involved in dyadic coping behaviour and at the impact of these behaviours on adaptation to the disease. Thus, Kayser and Acquati (2019) showed the significant role that relational mutuality plays in dyadic coping by associating it with the positive coping of both breast cancer patients and their partners and associating it negatively with avoidance in both partners. Another aspect of interest was the effect of dyadic coping on the couple’s relationship. Pankrath et al. (2016), Badr et al. (2010), Rottmann et al. (2015), and Ștefănuț et al. (2021b) found that whilst positive dyadic coping is significantly positively associated with the level of couple satisfaction, with a better dyadic adjustment, and with higher relationship quality, negative dyadic coping is associated with low levels of couple satisfaction, poorer dyadic adjustment, and lower relationship quality. Other effects of dyadic coping that were studied were concerned with depression, anxiety and supportive care needs. Thus, the higher the level of positive delegated coping of breast cancer patients towards life partners, the higher the level of depressive symptoms of the patients and their life partners. By contrast, the higher the level of delegated coping of life partners towards patients, the lower the level of depressive symptoms of the life partners (Rottmann et al., 2015). Regan et al. (2014) found that the use of supportive dyadic coping by prostate cancer patients and their life partners is not associated with patient depression and anxiety but is associated with life partner depression and anxiety. In addition, the perception of negative dyadic coping in which the life partners engage is also associated with their depression and anxiety. In their investigation, Weißflog et al. (2017) found that a high perception of negative dyadic coping by the life partner is associated with higher supportive care needs of both patients and their life partners. The same was found for communicating one’s stress and supportive care needs but only for patients. Regarding QoL, a longitudinal study by Ernst et al. (2016) shows that the dyadic coping of patients with haematological cancer and that of their life partners both affect their subsequent psychological quality of life. Also, both the dyadic coping of patients with haematological cancer and that of their life partners affect the subsequent mental and physical quality of life of partners. Considering the association of the constructs included in the model both with psychological variables at the individual and couple level, considering that the model proved applicable for different types of couples (young, middle-aged, elderly, heterosexual or not; Bodenmann et al., 2017) and dyadic coping repeatedly predicted the well-being of the partner (Falconier et al., 2015), the authors chose the Systemic Transactional Model as a theoretical foundation for the present study.

To the authors’ knowledge, of all studies based on the Systemic Transactional Model, this paper is the first to set out to examine the relationship between the perception of disease and dyadic coping. The Transactional Stress Theory of Lazarus and Folkman (1984) proposes a process-oriented approach to stress and coping. They consider that the context in which the stressful event occurs is extremely important because coping appears as a response to the psychological and environmental demands specific to this situation. In this way, their theory differs from trait-oriented approaches in which coping is considered a characteristic of the person and the variations of the context have a low importance. Cognitive appraisal is defined as the process by which the person evaluates whether a certain event is relevant for his well-being. Thus, primary appraisal refers to the consequences that the respective event could have on values, goals, personal well-being or on the well-being of a loved one. In secondary appraisal, the person evaluates whether something can be done to prevent the detected threat or to increase the potential benefits, depending on the situation. Primary and secondary appraisals converge to determine if the respective event is included in one of the threat or challenge categories. Threat implies the possibility of a future loss, and challenge reflects the anticipation of a gain or growth as a result of the experience (Folkman et al., 1986). In addition, Lazarus recognises that centrality, that is, the perceived importance of the event for the well-being of the person (Lazarus, 1984), plays an important role in the stress process. Coping is defined as the cognitive or behavioural effort that the person makes to manage various external or internal demands that are evaluated as exceeding his personal resources. Therefore, this study will aim to analyse, successively, the relationships between these components of perception of disease and the components of dyadic coping.

To the authors’ knowledge, there is one longitudinal study (Ernst et al., 2016) based on the Transactional Systemic Model that investigates the relationship between dyadic coping and the quality of life of patients with haematological cancer and their life partners. By contrast with that study, which looked at a population facing the specific difficulties of haematological cancer regardless of the phase of the disease, this study focuses on a population required to cope with the difficulties associated with the active phase of the treatment, such as the treatment itself, its possible side effects, the multiple interactions with medical staff, and the hospital setting itself. Thus, whilst the earlier (Ernst et al., 2016) study had around a quarter of patient participants (28%) who were in the active stage of treatment, this study aimed to include only patients in the active phase of treatment. The analysis considered three aspects of the quality of life of patients and their partners: global health/QoL, the person’s functioning, and the symptomatology felt. Therefore, the study investigated, successively, the relationships between the components of dyadic coping and these aspects of QoL. This cut-out at the level of dyadic coping as well as at the level of quality of life allows the identification of the truly beneficial components of dyadic coping as well as the aspects of quality of life on which they have an effect. This identification will allow future couple interventions to focus especially on specific coping behaviours, depending on the aspect of quality of life that is to be improved.

A further element of interest brought by this study is the fact that it intended to investigate the aforementioned relationships amongst the Romanian population. Most studies have been done on western samples, without offering a perspective of cultural background (Vintilă et al., 2020), whilst this population has up until now been little studied in terms of coping with cancer, whether on the part of patients or on that of their life partners. Even if for the young generations this situation is changing, the middle-aged and older generations predominantly present the profile of the collectivist individual in which the person tends to define himself in relation to others (David, 2015). In the context of a serious disease such as cancer, this fact can be reflected in the support offered by the family to the sick person, but at the same time it can also be reflected in the adoption of a less open communication in order not to reach conflict, embarrassment or not to create difficulties for the other (Merkin et al., 2014). Also, this collectivist profile of the individual can be reflected in providing care for the sick family member without considering their own needs, leading to negative consequences on the well-being of the caregiver.

The specific hypotheses reflected the actor and partner effects for the variables mentioned above. For easier reading, in the continuation of the manuscript, we will use the notions of “patient” and “life partner”/“caregiver” to denote the roles within couples and we will use the notions of “actor” and “partner” to refer to the elements of the APIM model.

*H1a*: the threat and centrality perceived by patients and their caregivers are negatively associated with the following components of dyadic coping: supportive dyadic coping by partner, delegated coping by self/by partner and common coping (actor and partner effects).

*H1b*: the threat and centrality perceived by patients and their caregivers is positively associated with negative dyadic coping (actor and partner effects).

*H2a:* the perception of disease as a challenge by patients and their caregivers is positively associated with the following components of dyadic coping: supportive dyadic coping by self/by partner, delegated coping by self/by partner and common coping (actor and partner effects).

*H2b*: the perception of disease as a challenge by patients and their caregivers is negatively associated with negative dyadic coping (actor and partner effects).

*H3a*: supportive dyadic coping by self, reported by patients and their caregivers is positively associated with general health/QoL and with QoL functionality (actor and partner effects).

*H3b*: supportive dyadic coping by self, reported by patients and their caregivers, is negatively associated with participants’ symptomatology (actor and partner effects).

*H4a*: supportive dyadic coping by partner, reported by patients and their caregivers, is positively associated with general health/QoL and with QoL functionality (actor and partner effects).

*H4b*: supportive dyadic coping by partner, reported by patients and their caregivers, is negatively associated with participants’ QoL symptomatology (actor and partner effects).

*H5a*: common coping reported by patients and their caregivers is positively associated with general health/QoL and QoL functionality (actor and partner effects).

*H5b*: common coping reported by patients and their caregivers is negatively associated with participants’ QoL symptomatology (actor and partner effects).

*H6a*: delegated coping by self is negatively associated with general health/QoL and QoL functionality reported by self (actor effect), and is positively associated with general health/QoL and QoL functionality reported by the other member of the couple (partner effect).

*H6b*: delegated coping by self is positively associated with one’s own QoL symptomatology (actor effect), and is negatively associated with the QoL symptomatology of the other member of the couple (partner effect).

*H7a*: delegated coping by partner is positively associated with one’s own general health/QoL and QoL functionality (actor effect), and is negatively associated with general health/QoL and QoL functionality reported by the other member of the couple (partner effect).

*H7b*: delegated coping by partner is negatively associated with one’s own QoL symptomatology (actor effect) and is positively associated with the QoL symptomatology of the other member of the couple (partner effect).

**Figure 1:**
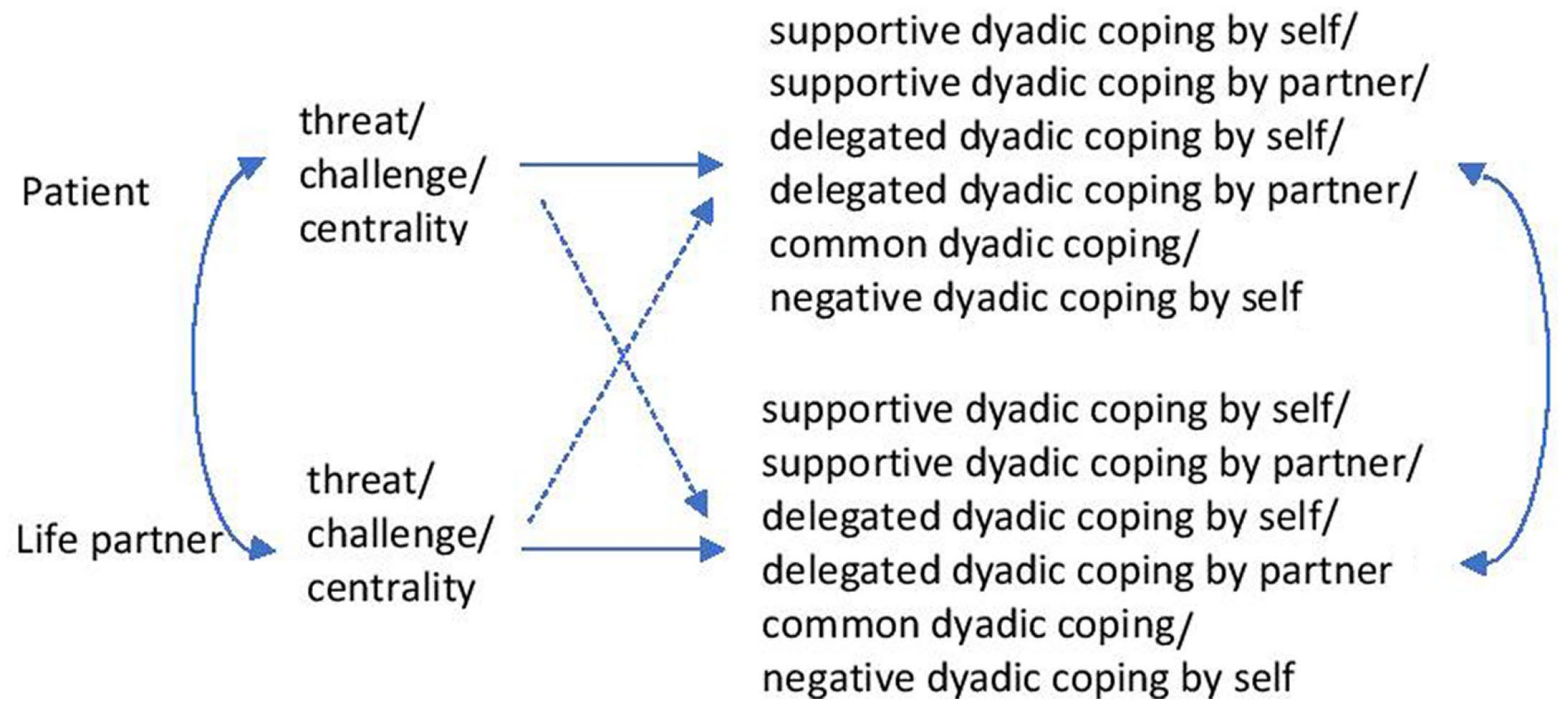
The relationships between the perception of the disease and the dyadic coping of one’s own and that of the partner.

**Figure 2:**
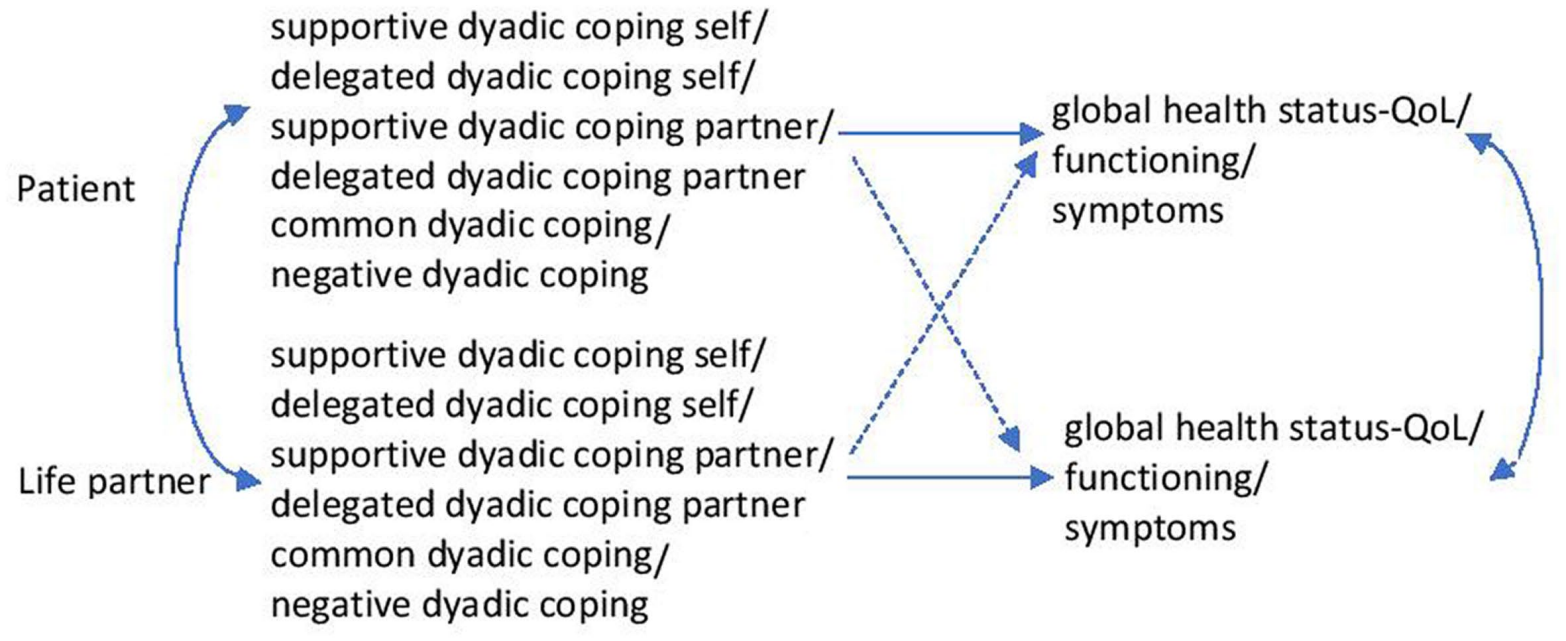
The relationships between dyadic coping and the quality of one’s own life and that of the partner.

Figures 1, 2 summarise the research hypotheses.

## Methods

### Procedure and participants

This cross-sectional study targeted oncological patients undergoing active treatment (radiotherapy, chemotherapy, hormone therapy, surgical intervention) and their life partners. Potential participant couples were recruited from patients coming in for treatment at the … Oncomed Outpatient Unit Timișoara, Romania. Recruitment of participants took place in the period April 2020–April 2021. Patients received an envelope that contained information about the study as well as instructions as to what steps to take if they wished to participate in it. The envelope also contained two other pre-stamped and pre-addressed envelopes holding the informed consent forms and the patient and life partner questionnaires. The instructions required participants to read and sign the consent forms, individually fill in the questionnaires, and then place them in these separate envelopes and send them to the research team. To encourage participation in the study, people who answered the questionnaires were given the opportunity to attend three free psychological couple counselling sessions.

To be eligible, couples had to meet the following criteria: (a) a member of the couple has been diagnosed with cancer and was undergoing active treatment for it; (b) both members of the couple had to be over the age of 18; (c) the two had to live together; (d) the life partner had to be the main person providing care and support to the patient; (e) both members of the couple had to speak and write fluently in Romanian. There was no inclusion criterion regarding the duration of the relationship of the participating couples.

This study has been approved by the Ethics Committee of the West University of Timisoara. In carrying out this study, the previously defined protocol was applied (Ştefănuţ et al., 2021a).

### Instruments

#### Socio-demographic and clinical data

Socio-demographic information, including age, gender, marital status, duration of the relationship, number of children, educational status, and occupational status, was collected from both patients and their life partners. Also, information regarding the medical characteristics, such as the initial location of the disease, its stage, treatment followed in the past, current treatment, the time elapsed since the diagnosis was found, and history of the disease in the family, was collected.

#### Perception of disease

The participants’ perception of the disease was evaluated using the *Threat*, *Challenge*, and *Centrality* scales of the *Stress Appraisal Measure* questionnaire (Peacock and Wong, 1990). This questionnaire was chosen because it measures cognitive appraisal as theoretically described (Carpenter, 2015) and was used in previous studies to evaluate the perception of other somatic (Rochette et al., 2007) or mental (Gorin et al., 2003) diseases. The *Threat* scale comprised 4 items and evaluated the extent to which the perceived event may involve a loss in the future. The *Challenge* scale included 4 items and measured the anticipation of a gain or growth as a result of the experience. *Centrality* is the scale that assessed the perceived importance of the event for the person’s well-being. An item on this questionnaire was: “*Does this situation have important consequences for me?*.” A 5-point Likert scale from 1 = not at all to 5 = extremely was used for answers. The study that defined the questionnaire reported for these scales a level of good internal consistency: for *Threat* between α = 0.65 and α = 0.75, for *Challenge* between α = 0.66 and α = 0.79, for *Centrality* between α = 0.84 and α = 0.90. To obtain the Romanian version of the questionnaire, the procedure of Beaton et al. (2000) was applied. Two independent translators, only one of whom was familiar with the questionnaire, translated it from English into Romanian. A third translator compared the two versions of the translation, resolved the differences and proposed a synthesis. Two other translators translated the resulting version back into English. In the next stage, all the translators involved as well as the authors of the article examined the different versions of the questionnaire (Romanian and English), debated the differences, and following obtaining the consensus, a final version in Romanian was proposed. The Cronbach α coefficients calculated for the present study were: for the *Threat* scale applied in the case of patients α = 0.85 and the case of partners α = 0.81; for the *Centrality* scale applied in the case of patients α = 0.84 and the case of partners α = 0.80. Because previous research found considerable variations for the Cronbach α coefficient associated with the *Challenge* scale: α = 0.66 in the last study defining the questionnaire (Peacock and Wong, 1990), α = 0.70 for the Turkish translation of the questionnaire (Durak and Senol-Durak, 2013) and α = 0.57 for its German translation (Delahaye et al., 2015), in the present study we checked the reliability of this scale using the split-half method. Thus, for the *Challenge* scale applied to patients, we obtained a value of 0.64 and for the same scale applied to their partners, we obtained a value of 0.65.

#### Dyadic coping

Dyadic Coping Inventory (Bodenmann, 2008), a validated tool that contains 37 items measuring both one’s coping behaviours (15 items), the perception of the partner’s coping behaviours (15 items), and the general satisfaction with this coping (2 items), was used to evaluate the dyadic coping of participants. The frequent use of this questionnaire in the dyadic coping analysis of oncological patients and their partners (Ștefănuț et al., 2021b) constituted the reason for its choice for use in the present study. The questionnaire covered the following dimensions: (a) *stress communication* (own stress—4 items; stress communicated by the partner—4 items), (b) *supportive dyadic coping* (own copying—5 items; supportive coping done by the partner—5 items), (c) *delegated coping* (own coping—2 items; delegated coping by the partner—2 items), (d) *negative dyadic coping* (own coping—4 items; negative coping by the partner—4 items), (e) *common coping* (5 items). An example of an item of this questionnaire was “*We try to cope with the problem together and search for ascertained solutions*.” Items were rated on a 5-point Likert scale from 1 = very rarely to 5 = very often. The values of Cronbach’s α coefficients for the subscales of the original questionnaire were satisfactory to very good, being between 0.71 and 0.92. This questionnaire was also available in Romanian (Rusu et al., 2016). For the Romanian version, the internal consistency of subscales was generally good, varying for women between α = 0.63 for their own delegated dyadic coping and α = 0.94 for evaluating dyadic coping, and for men varying between α = 0.63 for supportive dyadic coping oriented to the partner’s problem and α = 0.92 for the evaluation of dyadic coping. Exceptions were the subscale own supportive dyadic coping oriented to the problem (α = 0.51 for women and α = 0.52 for men) and the subscale common dyadic coping oriented to emotion (α = 0.51 for women). Since these subscales only contained 2 items, Cronbach α was equivalent to their correlation, and in this case, r > 0.5 which indicated a high correlation. The Cronbach α coefficients calculated for the sample included in this study ranged between α = 0.63 and α = 0.91. The scale *Delegated Dyadic Coping by Oneself* was an exception. Since the Cronbach α coefficient is not the best method of checking reliability when the number of items is small, for this scale, which contains only 2 items, we calculated the correlation coefficient and found it to be moderately statistically significant.

#### Quality of life

The European Organisation for Research and Treatment of Cancer QLQ-C30 questionnaire (Aaronson et al., 1993) was used to assess participants’ quality of life. This is a valid tool designed to measure the quality of life associated with health in cancer patients. The fact that this is the most used questionnaire for cancer patients in Europe and is also frequently used throughout the world (Fayers and Bottomley, 2002) determined its use for the evaluation of the participants in this study. The questionnaire contains 30 items grouped into 3 subscales that assessed *General health/QoL* (2 items), *the person’s Functioning* (15 items) and *Symptomatology* (13 items). An example of an item included in this questionnaire is “*Did you feel depressed?*.” Except for items corresponding to general health/QoL and which were evaluated on a 7-point Likert scale from 1 = very poor to 7 = excellent, the other items were evaluated on a 4-point Likert scale from 1 = not at all to 4 = very much. For the English version, all scales demonstrated a high internal consistency with Cronbach’s alpha coefficient > 0.7 (Bjordal et al., 2000). Versions available for other populations have also demonstrated good internal consistency. Thus, Cronbach’s α coefficients for the Italian version fell within the range of 0.64–0.90 (Apolone et al., 1998) and Cronbach’s α coefficients for the Kenyan version were higher than 0.70 (Davda et al., 2021). The study used the Romanian version of the questionnaire provided by The European Organisation for Research and Treatment of Cancer. The Cronbach α coefficients calculated for the present study were: for the *Symptomatology* scale applied in the case of patients α = 0.87 and the case of partners α = 0.88; for the *Functioning* scale applied to patients α = 0.89 and in the case of partners α = 0.85; for the *Global Health Status/QoL* scale applied in the case of patients α = 0.94 and the case of partners α = 0.80.

### Statistical methods

The APIMPowerR software tool was used to estimate the number of dyads that needed to be included in the study and resulted that, for this research, data needed to be collected from 131 patient-caregivers dyads.

The average difference in the perception of disease, dyadic coping and quality of life of patients and their caregivers was tested using paired *t-*tests.

The analytical strategy was based on modelling through structural equations and on the Actor-Partner Interdependence Model (APIM). For the estimation of the model parameters, modelling using structural equations was used. Multiple models were run successively. The patient’s and the caregiver’s perceptions of the disease (threat, challenge, centrality) were exogenous variables and dyadic coping components (supportive coping by oneself/by partner, delegated coping by oneself/by partner, common coping, negative dyadic coping) were endogenous variables. Separate analyses were run for each combination of these exogenous and endogenous measures (Figure 1). In other models dyadic coping components (supportive coping by oneself/by partner, delegated coping by oneself/by partner, common coping, negative dyadic coping) were exogenous variables and quality of life components (general health/QoL, QoL functionality of the person, symptomatology) were endogenous variables. Also, separate analyses were run for each combination of these exogenous and endogenous variables (Figure 2). The gender and age of participants, type of cancer, and stage of the disease were regarded as covariates. The Actor-Partner Interdependence Model was used to analyse whether patients’ and caregivers’ perception of the disease was associated with one’s dyadic coping (actor effect) or with the partner’s dyadic coping (partner effect). This model was also used to analyse to what extent the dyadic coping of patients and caregivers was associated with one’s quality of life (actor effect) or with the partner’s quality of life (partner effect). These analyses were performed successively. The SPSS v20 and R software (R Core Team, 2014) were used to perform these analyses.

## Results

### Participants characteristics

One hundred and thirty eight dyads formed of cancer patients and their life partners participated in the study. The average age of both patients and caregivers was 58 (M = 58.1, SD = 13.19; M = 58.51, SD = 13.99). The majority (87.7%) of dyads were married couples and the average length of their relationship was 30 years (M = 30.32, SD = 15.52). 45.7% of patients in the study were facing a diagnosis of breast cancer, and for 46.4% the disease was in stage IV. Participant data is summarised in Table 1.

**Table 1:** Participant characteristics.

	Patients (*N* = 138)	Life partners (*N* = 138)
Age (mean)	58.1 (SD = 13.19)	58.51 (13.99)
Length of relationship (mean)	30.32 (SD = 15.52)	
**Gender**		
Female	94 (68.1%)	44 (31.9%)
Male	44 (31.9%)	94 (68.1%)
**Marital status**		
Married	121 (87.7%)	
Living together	17 (12.3%)	
**Educational level**		
Middle school	22 (15.9%)	27 (19.6%)
High school	49 (35.5%)	35 (25.4%)
Vocational school	25 (18.1%)	34 (24.6%)
First degree	36 (26.1%)	38 (27.5%)
Master’s	6 (4.3%)	2 (1.4%)
Doctorate	-	2 (1.4%)
**Occupational status**		
Employed	38 (27.5%)	49 (35.5%)
Unemployed	5 (3.6%)	6 (4.3%)
Businessman/woman	3 (2.2%)	3 (2.2%)
Retired	92 (66.7%)	79 (57.2%)
**Type of cancer**		
Breast	63 (45.7%)	
Gynaecological	16 (11.6%)	
Prostate	4 (2.9%)	
Gastro-intestinal	11 (8%)	
Lung	19 (13.8%)	
Head area	6 (4.3%)	
Colon	12 (8.7%)	
Pancreatic	3 (2.2%)	
Other	4 (2.9%)	
**Current stage**		
I	7 (5.1%)	
II	38 (27.5%)	
III	29 (21%)	
IV	64 (46.4%)	
**Previous treatment**		
Radiotherapy	5 (3.6%)	
Chemotherapy	11 (8%)	
Surgical	23 (16.7%)	
Hormone therapy	5 (3.6%)	
Several kinds of treatment	85 (61.6%)	
No treatment	9 (6.5)	
**Current treatment**		
Radiotherapy	5 (3.6%)	
Chemotherapy	68 (49.3%)	
Surgical	3 (2.2%)	
Hormone therapy	52 (37.7%)	
Other	10 (7.2%)	
Length of time since diagnosis (months)	29.55 (SD = 33.94)	
**Trajectory of the disease**		
New diagnosis	64 (46.3%)	
Recurrence	74 (53.7%)	
**Family history of cancer**		
No	103 (74.6%)	
Yes	35 (25.4%)	

### Description of statistics and correlations between relevant variables

There were no statistically significant differences in the way patients and their caregivers perceived the disease from the point of view of the threat, challenge, or centrality (Table 2). Patients reported levels of supportive dyadic coping by the partner and delegated dyadic coping by the partner that were significantly higher than those reported by caregivers (*t* = 4.33, *p* < 0.001; *t* = 5.02, *p* < 0.001). The same kind of result was also obtained for negative dyadic coping by partner (*t* = 3.04, *p* < 0.01) and for negative dyadic coping by oneself (*t* = 2.25, *p* < 0.05). The symptomatology of patients was significantly more accentuated (*t* = 9.64, *p* < 0.001) and their QoL functionality significantly more negatively impacted (*t* = 7.43, *p* < 0.001) than was the case for caregivers, and the global health/QoL level reported by patients was significantly lower than that reported by their caregivers (*t* = 7.21, *p* < 0.001; Table 2).

**Table 2:** Patient and partner scores for perception of disease, dyadic coping and quality of life.

Variable	Patients		Life partners		*t*	*p*
	M	SD	M	SD		
**Perception of disease**						
Threat	11.45	4.27	11.38	4.04	0.19	0.84
Centrality	12.99	4.05	12.87	3.78	0.35	0.72
Challenge	11.47	3.09	11.13	3.15	1.22	0.22
**Dyadic coping**						
Supportive dyadic coping by oneself SDCO	18.54	3.93	18.65	3.93	0.39	0.69
Supportive dyadic coping by partner SDCP	19.28	4.51	17.96	4.81	4.33	0.00^***^
Delegated dyadic coping by oneself DDCO	6.88	1.81	7.15	1.83	1.45	0.14
Delegated dyadic coping by partner DDCP	7.56	2.13	6.67	2.06	5.02	0.00^***^
Common/Shared dyadic coping CDC	18.41	4.96	18.09	5.18	1.27	0.20
Negative dyadic coping by oneself NDCO	14.83	3.64	14.23	3.56	2.25	0.02^*^
Negative dyadic coping by partner NDCP	14.64	3.76	13.82	3.62	3.04	0.003^**^
**Quality of life**						
Functionality	32.67	9.02	26.52	7.65	7.43	0.00^***^
Symptoms	26.59	7.70	20.30	6.79	9.64	0.00^***^
Global health	8.97	2.90	10.60	2.28	7.21	0.00^***^

^*^p < 0.05, ^**^p < 0.01, ^***^p < 0.001.

Statistically significant correlations were obtained between all the components of perception of disease, dyadic coping, and quality of life of patients and the corresponding components reported by their caregivers (Table 3).

**Table 3:** Correlations between components of perception of disease, dyadic coping and quality of life.

	Thr 1	Cen 1	Cha1	SDCO1	SDCP1	DDCO1	DDCP1	CDC1	NDCO1	NDCP1	Fct 1	Sym 1	Hlt 1	Thr 2	Cen 2	Cha2	SDCO2	SDCP2	DDCO2	DDCP2	CDC 2	NDCO 2	NDCP 2	Fct 2	Sym 2	Hlt 2
Thr1	1																									
Cen1	0.76^**^	1																								
Cha1	−0.03	0.18^*^	1																							
SDCO1	−0.33^**^	−0.21^*^	0.28^**^	1																						
SDCP1	−0.35^**^	−0.28^**^	0.15	0.67^**^	1																					
DDCO1	−0.23^**^	−0.26^**^	0.12	0.59^**^	0.41^**^	1																				
DDCP1	−0.20^*^	−0.14	0.15	0.58^**^	0.77^**^	0.36^**^	1																			
CDC1	−0.36^**^	−0.25^**^	0.27^**^	0.73^**^	0.73^**^	0.47^**^	0.65^**^	1																		
NDCO1	−0.07	0.06	0.24^**^	0.19^*^	0.15	0.12	0.15	0.24^**^	1																	
NDCP1	−0.15	0.06	0.32^**^	0.26^**^	0.37^**^	0.10	0.32^**^	0.32^**^	0.65^**^	1																
Fct1	0.43^**^	0.46^**^	−0.11	−0.11	−0.13	−0.08	0.01	−0.15	−0.07	−0.10	1															
Sym1	0.35^**^	0.34^**^	0.03	−0.13	−0.16	−0.09	−0.01	−0.18^*^	0.05	−0.03	0.79^**^	1														
Hlt1	−0.44^**^	−0.38^**^	0.09	0.16	0.17^*^	0.18^*^	0.11	0.20^*^	0.01	0.07	−0.57^**^	−0.50^**^	1													
Thr2	0.56^**^	0.51^**^	−0.04	.-0.23^**^	−0.17^*^	−0.17^*^	−0.03	−0.16	0.06	0.00	0.39^**^	0.35^**^	−0.35^**^	1												
Cen2	0.41^**^	0.52^**^	0.07	−0.08	−0.08	−0.16	0.01	−0.05	0.15	0.15	0.35^**^	0.24^**^	−0.33^**^	0.75^**^	1											
Cha2	−0.02	0.11	0.45^**^	0.13	0.17^*^	−0.04	0.16	0.28^**^	0.12	0.23^**^	−0.07	0.00	0.007	0.14	0.22^**^	1										
SDCO2	−0.26^**^	−0.13	0.21^**^	0.61^**^	0.66^**^	0.40^**^	0.56^**^	0.63^**^	0.25^**^	0.36^**^	−0.10	−0.15	0.17^*^	−0.09	−0.02	0.18^*^	1									
SDCP2	−0.43^**^	−0.28^**^	0.33^**^	0.68^**^	0.71^**^	0.43^**^	0.57^**^	0.77^**^	0.26^**^	0.33^**^	−0.19^*^	−0.16^*^	0.27^**^	−0.33^**^	−0.17^*^	0.24^**^	0.67^**^	1								
DDCO2	−0.25^**^	−0.17^*^	0.04	0.39^**^	0.49^**^	0.30^**^	0.49^**^	0.43^**^	0.17^*^	0.25^**^	−0.04	−0.05	0.12	−0.05	−0.01	0.18^*^	0.68^**^	0.48^**^	1							
DDCP2	−0.33^**^	−0.24^**^	0.26^**^	0.43^**^	0.44^**^	0.40^**^	0.50^**^	0.54^**^	0.06	0.15	−0.14	−0.07	0.28^**^	−0.32^**^	−0.21^*^	0.16^*^	0.34^**^	0.64^**^	0.23^**^	1						
CDC2	−0.37^**^	−0.26^**^	0.24^**^	0.64^**^	0.72^**^	0.40^**^	0.58^**^	0.84^**^	0.25^**^	0.31^**^	−0.23^**^	−0.22^**^	0.30^**^	−0.22^**^	−0.12	0.26^**^	0.72^**^	0.82^**^	0.53^**^	0.56^**^	1					
NDCO2	−0.08	0.07	0.11	0.24^**^	0.36^**^	0.14	0.33^**^	0.34^**^	0.62^**^	0.64^**^	0.04	−0.01	−0.05	0.03	0.22^**^	0.17^*^	0.34^**^	0.29^**^	0.28^**^	0.07	0.24^**^	1				
NDCP2	−0.14	0.07	0.23^**^	0.29^**^	0.28^**^	0.13	0.17^*^	0.33^**^	0.56^**^	0.62^**^	−0.04	−0.007	0.03	−0.002	0.14	0.21^*^	0.35^**^	0.37^**^	0.26^**^	0.07	0.28^**^	0.68^**^	1			
Fct2	0.16	0.09	−0.09	−0.10	−0.07	−0.03	−0.01	−0.01	−0.15	−0.18^*^	0.33^**^	0.38^**^	−0.16	0.39^**^	0.21^*^	0.16	−0.11	−0.16^*^	−0.06	−0.05	−0.10	−0.19^*^	−0.12	1		
Sym2	0.07	0.10	0.05	−0.13	−0.09	−0.04	−0.05	−0.02	−0.04	−0.07	0.24^**^	0.44^**^	−0.12	0.24^**^	0.16^*^	0.22^**^	−0.19^*^	−0.10	−0.09	−0.08	−0.10	−0.10	−0.03	0.76^**^	1	
Hlt2	−0.21^**^	−0.11	0.09	0.16	0.08	0.03	0.10	0.14	0.10	0.12	−0.34^**^	−0.37^**^	0.49^**^	−0.29^**^	−0.23^**^	−0.09	0.20^*^	0.19^*^	0.20^*^	0.03	0.24^**^	0.05	0.001	−0.55^**^	−0.48^**^	1

^*^p < 0.05, ^**^p < 0.01, ^***^p < 0.001. Thr, Cen, Cha, scores correspond to the Threat, Centrality, Challenge subscales of the Stress Appraisal Measure questionnaire; SDCO, SDCP, DDCO, DDCP, CDC, NDCO, NDCP, subscales Supportive dyadic coping by oneself, Supportive dyadic coping by partner, Delegated dyadic coping by oneself, Delegated dyadic coping by partner, Common dyadic coping, Negative dyadic coping by oneself, Negative dyadic coping by partner. Fct, Sy, Hlt, subscales Functionality, Symptoms and Global health/QoL of the European Organisation for Research and Treatment of Cancer questionnaire QLQ C30. Variables corresponding to patients are marked 1. Variables corresponding to partners are marked 2.

### Effects of perception of the disease by participants on their own and their partners’ dyadic coping

Perception of the threat of the disease by patients had a significant negative effect on all positive forms of dyadic coping and negative dyadic coping by the partner, both for themselves (actor effect) and their caregivers (partner effect). Also, the patients’ perception of centrality had a significant negative effect on all positive forms of dyadic coping for themselves (actor effects). A statistically significant partner effect was obtained for the influence of the patients’ perception of centrality on the supportive dyadic coping by partner, on the common dyadic coping, and the delegated dyadic coping by oneself. So, the results obtained support the H1a hypothesis for patients but not for caregivers and the H1b hypothesis is not supported. Patients who perceived the disease more as a challenge became involved in significantly more common dyadic coping behaviours, supported their caregivers significantly more and took on significantly more of their tasks (actor effects). Also, it was found that the perception of the disease as a challenge by caregivers had a significant positive impact on their common dyadic coping (actor effect) but also on patients’ common dyadic coping (partner effect). The obtained results support part of the relationships proposed by hypothesis H2a, but this hypothesis is not supported in its totality. The H2b hypothesis is not supported by the results.

Effects of perception of the disease on own and one partner’s dyadic coping components are summarised in Table 4.

**Table 4:** Influence of perception of the disease on dyadic coping of patients and their partners.

Patients							
	SDCO	SDCP	DDCO	DDCP	CDC	NDCO	NDCP
**Perceived by patients**							
Threat	−3.08^**^	−3.87^***^	−2.02^*^	−2.77^**^	−4.19^***^	−1.64	−2.17^*^
Challenge	3.03^**^	1.02	1.97^*^	1.07	2.01^*^	2.53^*^	3.02^**^
Centrality	−2.38^*^	−3.47^***^	−2.58^*^	−2.11^*^	−3.25^***^	−0.21	−0.23
**Perceived by partners**							
Threat	−0.67	0.38	−0.62	1.24	0.70	1.54	1.23
Challenge	0.05	1.37	−1.35	1.25	2.27^*^	0.17	1.20
Centrality	0.33	0.99	−0.35	1.24	1.16	1.72	1.73
**Partners**							
	**SDCO**	**SDCP**	**DDCO**	**DDCP**	**CDC**	**NDCO**	**NDCP**
**Perceived by patients**							
Threat	−3.05^**^	−3.86^***^	−3.30^***^	−2.28^*^	−3.86^***^	−1.49	−2.03^*^
Challenge	1.80	3.18^***^	−0.57	2.61^**^	1.76	0.40	1.86
Centrality	−1.72	−2.78^**^	−2.37^*^	−1.84	−2.8^**^	−0.55	−0.006
**Perceived by partners**							
Threat	0.74	−1.47	1.29	−2.09^*^	−0.17	1.19	1.13
Challenge	1.22	1.26	2.19^*^	0.65	2.06^*^	1.72	1.48
Centrality	0.69	−0.33	1.10	−1.25	0.14	2.60^**^	1.47

^*^p < 0.05, ^**^p < 0.01, ^***^p < 0.001. SDCO, Supportive dyadic coping by oneself; SDCP, Supportive dyadic coping by partner; DDCO, Delegated dyadic coping by oneself; DDCP, Delegated dyadic coping by partner; CDC, Common dyadic coping; NDCO, Negative dyadic coping by oneself; NDCP, Negative dyadic coping by partner.

### Effects of participants’ dyadic coping on own and partners’ quality of life components

When patients report that caregivers engage in more negative dyadic coping behaviours, caregivers report significantly higher levels of global health/QoL (partner effect). Supportive dyadic coping by the partner, delegated dyadic coping by oneself and common dyadic coping had a significant positive actor effect on caregivers’ global health/QoL. The obtained results support part of the relationships proposed by hypotheses H4a and H5a, but these hypotheses are not supported in their totality. We also noted that the symptomatology of patients and caregivers is not significantly influenced either by the dyadic coping of patients or by the dyadic coping of their caregivers.

From the results obtained we can state that they do not sustain hypotheses H3a, H3b, H4b, H5b, H6a, H6b, H7a, and H7b.

The effects of dyadic coping on the components of own and partners’ quality of life are summarised in Table 5.

**Table 5:** Influence of dyadic coping on patients’ and their partners’ quality of life.

Patients			
	Functioning	Symptoms	Global health/QoL
**Reported by patients**			
SDCO	−0.66	−0.67	0.94
SDCP	−0.06	−0.75	−0.33
DDCO	−0.88	−0.98	1.88
DDCP	1.23	0.24	−0.47
CDC	0.96	0.20	−1.26
NDCO	−1.44	0.85	0.73
NDCP	−1.22	−0.43	0.88
**Reported by partners**			
SDCO	−0.60	−1.05	1.04
SDCP	−1.56	−0.87	2.57^*^
DDCO	−0.27	−0.28	0.88
DDCP	−2.10^*^	−0.82	3.30^***^
CDC	−2.35^*^	−1.64	3.13^**^
NDCO	1.27	−0.63	−0.91
NDCP	0.38	0.21	−0.25
**Partners**			
	**Functioning**	**Symptoms**	**Global health/QoL**
**Reported by patients**			
SDCO	−0.53	−0.16	0.58
SDCP	0.69	−0.42	−0.93
DDCO	−0.24	−0.21	−0.35
DDCP	0.18	−0.23	1.22
CDC	1.42	1.39	−1.34
NDCO	−0.44	0.30	1.10
NDCP	−1.63	−0.76	1.97^*^
**Reported by partners**			
SDCO	−0.71	−1.78	1.63
SDCP	−1.91	−0.54	2.31^*^
DDCO	−0.62	−1.05	2.41^*^
DDCP	−0.59	−0.69	−0.24
CDC	−1.84	−1.86	2.71^**^
NDCO	−1.56	−1.15	−0.15
NDCP	−0.11	0.15	−1.22

^*^p < 0.05, ^**^p < 0.01, ^***^p < 0.001. SDCO, Supportive dyadic coping by oneself; SDCP, Supportive dyadic coping by partner; DDCO, Delegated dyadic coping by oneself; DDCP, Delegated dyadic coping by partner; CDC, Common dyadic coping; NDCO, Negative dyadic coping by oneself; NDCP, Negative dyadic coping by partner.

## Discussion

The study aimed to examine the relationship between dyadic coping and (1) disease perception and (2) quality of life of a sample of cancer patients and their life partners. Our hypotheses were formulated to express the fact that disease perception would influence own and partner’s dyadic coping and the fact that dyadic coping would influence own and life partner’s quality of life. By applying the Actor-Partner Interdependence Model for statistical analysis of data collected from the 138 patient-partner dyads it was found that some relationships included in the hypotheses were supported, but no hypothesis was supported in its entirety.

No previous studies known to the authors have analysed the influence of perception of the disease (its threatening, challenging, and centrality) on dyadic coping. If we look at the first of these components, the perception of the disease as threatening by patients had a significant negative effect on all the positive forms of own and also of life partners’ dyadic coping, suggesting that the more threatening the disease is perceived by patients as being, the more pronounced a withdrawal from the relationship between the two takes place. Where patients are concerned, one possible explanation for this disengagement could be the fact that they are devoting their resources to coping with the medical aspects of their illness. The higher the disease threat as perceived by caregivers, the lower was the level of delegated coping reported by them as having been achieved by the patients, which is not surprising given that in a situation of serious illness the caregivers are the ones who take over patients’ normal tasks and not the other way round.

A further component of disease perception analysed in this study was its challenging nature. Results show that the more conscious patients are of the possibility of some gain or growth arising from the experience of the disease, the more they engage in behaviours specific to positive dyadic coping. Similarly, the more caregivers perceive the disease as a challenge, the more they engage in behaviours that support patients and in common coping. This finding is not surprising if we remember that one of the positive aspects mentioned by people who were facing cancer and by their life partners is an improvement in their relationships, along with increased confidence in their resources, a heightened appreciation of life, and the discovery of new opportunities (Manne et al., 2004). Therefore, both in practice and in the interventions targeted by future research, it can be aimed at encouraging couples to perceive the disease more as a challenge.

Another aspect of the perception of the disease analysed in this research was its centrality. Results demonstrated that the more patients perceive that the disease may have serious negative long-term consequences for their well-being, the fewer behaviours associated with positive forms of dyadic coping they carry out and the fewer such behaviours they perceive their caregivers as carrying out. Future studies could analyse whether perceived control over the disease influences on this relationship.

When caregivers report more coping behaviours performed jointly with the patients and higher practical support from them, the patients perceived that QoL functionality was more negatively impacted but described their health/QoL as being better. A possible explanation for this could be that the help provided by patients with a range of specific tasks, along with involvement in shared activities, can result in the cancer sufferers becoming physically exhausted whilst at the same time the achievement of these tasks appears to be viewed by them as a sign of good health and a good QoL. Thus, taking part in these kinds of behaviours could help to restore a feeling of being capable of achieving things and of being useful in the relationship. Moreover, the systematic review by Traa et al. (2015) highlighted that cancer couples characterised by a high level of supportive behaviours and active engagement present higher levels of relationship functioning. Thus, practitioners and researchers who aim to increase the QoL and relationship functioning of couples facing cancer could encourage patients to be more active, and to participate in joint activities without reaching physical exhaustion.

A further effect observed was the significant positive influence of the practical support given by caregivers on their general state of health/QoL. This can be accounted for by the fact that although caring for a patient suffering from cancer can be stressful, it can at the same time be gratifying. This idea is consistent with what has been highlighted in another research. For example, Manne et al. (2004) showed that both patients and significant others reported posttraumatic growth a year and a half after the diagnosis of breast cancer. The research results have also shown that supportive dyadic coping provided by patients and reported by caregivers had the effect of improving caregivers’ global health status/QoL. These results underline the importance of mutual emotional support in couples confronting cancer and make patients contributors to the couple’s wellbeing, not mere receivers of care and support from their life partners.

The present study contributes to a detailed understanding of how disease perception and dyadic coping can promote a better quality of life in cancer patients and their life partners. The meta-analysis carried out by Badr and Krebs (2013) highlighted that couple-based interventions have a beneficial effect on the QoL of both patients and their partners and our results are of potential use to researchers and clinicians as they seek to develop interventions that can improve how partners cope together with the disease. For example, future interventions could encourage couples to see the disease as a challenge that brings with it possible beneficial consequences, including improving interpersonal relationships, strengthening one’s ability to face up to hardships, developing a greater appreciation of life, discovering new opportunities, and spiritual growth by expressing their shared values and beliefs which are keys of reading and interpreting of couple’s reality (Rusu and Turliuc, 2011). Future initiatives could also be aimed at helping couples facing cancer to understand that the disease is a shared challenge and that the emotional and practical support they give each other helps them cope more easily with this difficult experience. Life partners could be encouraged to take over responsibility for activities patients can no longer perform whilst at the same time patients too could be encouraged to play an active role, to give their caregivers emotional support and, to the extent to which their state of health permits it, to carry out a range of tasks. Another aspect that could be included in future interventions is encouraging and developing skills for shared problem-solving and identifying of relaxing shared activities. These proposals are consistent with what was found in previous research, namely the fact that the functioning of couples in an oncological context depends on how well they have integrated cancer in their lives (Manne and Badr, 2008).

The results of this study highlighted significant associations between dyadic coping and quality of life. Framed in the broader context of the literature, they contribute to sustaining the fact that dyadic coping is significantly associated with individual psychological variables (quality of life, depression, anxiety) but also with interpersonal psychological variables at the couple level (dyadic adjustment, relationship satisfaction). This finding recommends the use of the Systemic Transactional Model as a theoretical foundation for future interventions dedicated to the psychological adjustment to the disease of couples facing cancer.

The study also has several limitations. First, the cross-sectional design employed means that it is not possible to see how the relationships highlighted developed over time. In addition, only components concerned with the initial evaluation of the disease were taken into consideration; secondary appraisal involving its controllable/uncontrollable nature was not included. Another limitation is that the study may contain only those couples who were willing to share their experiences and the sample may not be a representative one. For example, Gouveia et al. (2022) showed that patients who wanted to participate in research presented several different psychological characteristics such as family satisfaction, social support and intimacy significantly higher than patients who refused to participate. Lastly, the fact that data was collected from just one centre may also impact the degree to which it is possible to generalise the results.

Regarding the future research directions opened up by this study, these could include looking at the influence of secondary appraisal on the relationship between primary appraisal of the disease and dyadic coping and at the possible moderating effect of intra-couple communication. Likewise, future studies could consider the analysis of the relationships between the perception of the disease, dyadic coping and the quality of life in the case of other diagnoses such as small renal masses for which it was found that patients face a reduction in the quality of life (Vartolomei et al., 2022). Also, future research could investigate if there are dyadic influences of the perception of the oncological disease on other variables of interest reported in the literature such as sleep disturbances.

## Conclusion

This study has highlighted new information regarding how couples cope with cancer by looking at the dyadic level. In clinical practice, this information can be used by healthcare providers to develop intervention strategies to improve the quality of life of cancer patients and their life partners.

## Data availability statement

The datasets presented in this study can be found in online repositories. The names of the repository/repositories and accession number(s) can be found at: osf.io: https://doi.org/10.17605/OSF.IO/6WK8A.

## Ethics statement

The studies involving human participants were reviewed and approved by the Ethics Committee of the West University of Timisoara, Romania (25621/0-1/16.06.2020 RCE2020-66). The patients/participants provided their written informed consent to participate in this study.

## Author contributions

AMȘ and MV contributed to all phases of the article. CG contributed to design phase and data collection. LMB, DG and CMO contributed to the collection of data. PS contributed to analysis of data. All authors contributed to the article and approved the submitted version.

## Funding

Open access publication fees received from West University of Timisoara.

## Conflict of interest

The authors declare that the research was conducted in the absence of any commercial or financial relationships that could be construed as a potential conflict of interest.

## Publisher’s note

All claims expressed in this article are solely those of the authors and do not necessarily represent those of their affiliated organizations, or those of the publisher, the editors and the reviewers. Any product that may be evaluated in this article, or claim that may be made by its manufacturer, is not guaranteed or endorsed by the publisher.
